# Population Declines of the Oriental Magpie *Pica serica* (Corvidae) in the Saga Plain, Japan: Relationships With Land Use Patterns, Nesting Sites, and Breeding Success

**DOI:** 10.1002/ece3.72193

**Published:** 2025-09-21

**Authors:** Takuho Nagafuchi, Yasue Bussaka, Makihiko Ikegami, Kao Tsuchiya, Makoto Tokuda

**Affiliations:** ^1^ Laboratory of Systems Ecology, Faculty of Agriculture Saga University Saga Japan; ^2^ Kanzaki City Saga Japan; ^3^ National Institute of Environmental Studies (NIES) Lake Biwa Office Shiga Japan; ^4^ The United Graduate School of Agricultural Sciences Kagoshima University Kagoshima Japan

**Keywords:** land‐use pattern, nest site, nesting success, oriental magpie, *pica serica*, population decline

## Abstract

Urban areas are hotspots of biological invasions where land use and other factors are associated with the success of non‐native species. Non‐native species alter their habits during the process of adapting to new environments, making them important subjects for studying organismal environmental adaptation. The Oriental Magpie *Pica serica* was introduced and established a substantial population in the Saga Plain, Japan, from the Korean Peninsula, probably by the early 17th century. As an adaptation to urbanization, this species has gradually begun to prefer to nest on utility poles rather than trees since around the late 1970s, but has declined its population since the 1990s. In this study, we hypothesized that the recent magpie decline is related to the changes in land use patterns and that magpie density affects its breeding success. We investigated the nesting density of magpies in the Saga Plain and compared the density with previous surveys. In addition, we surveyed the breeding success in areas with low and high magpie densities. As a result, the magpie densities rapidly decreased from 2008 to 2019, whereas nesting rates on artificial structures increased significantly. In our analysis, building and farmland areas themselves had a positive effect on magpie density. Over the recent decade, the building area has increased, whereas the farmland has decreased in our census sites. Then the negative impacts of the farmland reduction were considered larger than the positive impacts of the increase in building areas. These suggest that urban developments critically affect the magpie decline. Breeding success rates were significantly higher in areas with high magpie density than in low‐density areas. In low‐density areas, success rates from egg hatch to fledging are significantly lower than in high‐density areas, suggesting that low‐density trends of magpies in most areas facilitate breeding failure and further negative impacts on the populations.

## Graphical abstract

The Oriental Magpie was introduced in the Saga Plain from the Korean Peninsula, probably by the early 17th century, and adapted to environmental changes. We revealed that the magpie population rapidly declined in these decades. Urban developments in farmland areas critically affected the decline, and breeding success rates were significantly higher in high‐density areas than in low‐density areas.

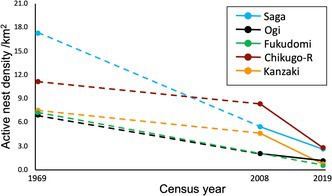

## 1 Introduction

Urbanization is a global trend, and human populations living in urban areas have increased from 30% in 1950 to 55% by 2018 (United Nations, Department of Economic and Social Affairs, Population Division 2019). As a result of urbanization and rural depopulation, the landscape and biodiversity gradients between urban and rural areas have become more pronounced (Xiao et al. 2016; Pal et al. 2019). One of the negative consequences of urbanization is the introduction and establishment of non‐native species, making the urban areas invasion hotspots (Gaertner et al. 2017). The invasion of non‐native organisms and their influences on ecosystems and biodiversity are a global concern today (Ricciardi et al. 2013; Simberloff et al. 2013; Blackburn et al. 2014; Crystal‐Ornelas and Lockwood 2020; Bacher et al. 2024). Although it is a well‐established fact that invasive alien species have various negative impacts on local biodiversity (Parker et al. 1999; Asner and Vitousek 2005; Pearse et al. 2019; Bacher et al. 2024), less is known about the population dynamics of these species in urban environments and the ecological or anthropogenic factors that facilitate their proliferation (Buenrostro and Hufbauer 2022; Carlon and Dominoni 2024).

Understanding how invasive species establish and thrive in novel environments requires examining their capacity to adapt to new ecological conditions. During the invasion process, many alien species undergo behavioral or physiological changes that enhance their survival and reproduction in urban settings (Alberti et al. 2016, 2017; Wang and Liu 2021). Exploring trait changes between their native and invaded areas could reveal critical driving factors creating novel interactions among organisms within these environments (Alberti et al. 2016, 2017). Investigating these adaptive responses is crucial for uncovering the mechanisms behind invasion success and for predicting future impacts on native ecosystems (Carroll 2007; Howard 2019).

The Oriental Magpie, *Pica serica* (Passeriformes: Corvidae), of which Japanese populations have been commonly mentioned as *P. pica* or *P. pica sericea* in pieces of literature (review in Eguchi 2016), is a resident bird distributed from southeast Russia to Myanmar, including the Korean Peninsula, Kyushu, east China, and Taiwan (Kryukov et al. 2017; Song et al. 2018; Gill et al. 2024). Now the population of northern Kyushu is distributed in areas at elevations of less than 100 m asl. in the Saga Plain and surrounding areas (Kubo 1971; Kubo and Matsuo 1972; Eguchi 2016). According to folklore, this population was originally brought back from Korea by the order of the founder of the Saga Clan at the end of the 16th century, during the battle of Bunroku and Keicho (between Japan and Korea), and was released in the territory in northern Kyushu (Eguchi and Kubo 1992; Eguchi 2016). Because of this background, protection orders were often issued by the ruling class of the Saga Clan in the early stages of the introduction (Eguchi and Kubo 1992) and their habitat in these areas was designated as a national monument of Japan in 1923 (https://bunka.nii.ac.jp/heritages/detail/164291), despite its non‐native status. Because of this conservation interest, changes in the distribution and abundance of magpies have been relatively well documented in the Saga Plain, which made the species a suitable organism to study the adaptation of birds to novel environments at a long timescale (Eguchi 2016).

The historical data showed that the magpie population in the Saga Plain has experienced three periods of density fluctuation since 1970 (Eguchi 2016): i.e., (1) a decreasing trend in the early 1970s, (2) a rapidly increasing trend in the late 1970s, and (3) a consistent decreasing trend since the 1990s. The first trend is thought to be due to a decrease in the amount of arable land following human population growth and urbanization, as well as the decrease in trees available for nesting. In the second trend, magpies frequently nest on utility poles rather than trees, and the change in this habit is considered to contribute to the population increase. In this term, the increase in the number of utility poles possessing horizontal splints probably facilitated nesting by magpies and thus contributed to the population growth of magpies in urban and suburban areas of the Saga Plain. In the third trend, foraging areas are considered to have decreased following the further progression of urbanization, since there has been a marked decline in areas with high utility pole densities and building rates (Saga Prefectural Board of Education 2014; Eguchi 2016).

In the Saga Plain, magpies are distributed mainly in arable land but not in forest areas (Kubo 1971). On the basis of the survey around 1990, the nesting density of magpies was higher in areas with more arable land (Eguchi 2016). However, a positive correlation was detected in the nesting density with the percentage of land used for buildings in the 2000s (Saga Prefectural Board of Education 2014; Eguchi 2016).

In this study, we hypothesized that the magpie populations continue to decline in the 2010s under conditions of further urbanization, the decline is related to the changes in land use patterns, and the magpie density affects its breeding success. To confirm the appropriateness of the hypotheses, we conducted field surveys of the magpie population in the Saga Plain in 2019 and compared the nesting density mainly with similar surveys conducted in 2008 (Bussaka 2009) and partly with those in 1969 (Kubo and Matsuo 1972). Then, we analyzed whether the land use patterns are related to the population changes of magpies. We also examined the breeding success of magpies between 2011 and 2013 in areas of high and low magpie densities in the Saga Plain.

## 2 Materials and Methods

### 2.1 Background on Magpies in the Saga Plain

To provide a context for the study area, we refer to Eguchi (2016), which summarizes the ecology of magpies in the Saga Plain. Magpies in the Saga Plain have shown notable changes in nesting behavior over the past several decades, particularly in response to urbanization. Although only about 5% of nests were built on artificial structures such as utility poles in the mid‐20th century, this proportion increased dramatically to over 90% by 2012. Since the late 1980s, magpies have preferred utility poles for nesting even when trees are available, suggesting a strong adaptation to human‐modified environments.

In areas other than Japan, magpies are generally known to build their nests on trees (Birkhead 1991), and recent surveys in urban environments also show strong tree‐nesting habits in both Europe (Šálek et al. 2021) and China (Xu et al. 2020). Thus, a strong nest‐site preference for man‐made structures of magpie populations in Kyushu is noteworthy and is a typical example of environmental adaptation to urbanization in birds.

In the Saga Plain, earlier studies reported relatively high nesting success rates (Eguchi 2016), comparable to those observed in magpie populations in Europe and North America (Birkhead 1991). However, in the survey between 1988 and 1993, the success rate was reported to have remarkably dropped to 18% (Eguchi and Takeishi 1997), which may also facilitate the magpie population decline in the Saga Plain.

### 2.2 Survey Site and Survey Plot

**FIGURE 1:**
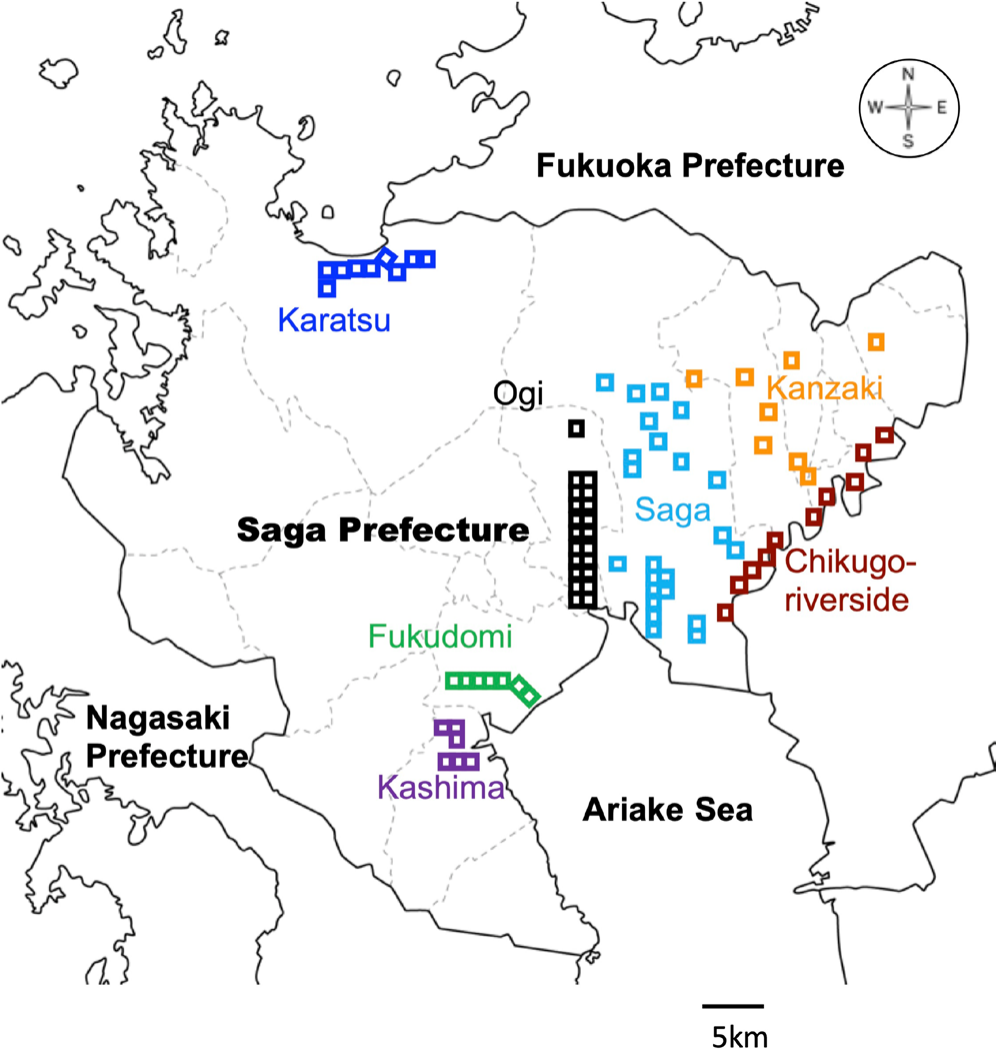
Map of census sites for the nesting density of magpies in Saga Prefecture, Kyushu, Japan. Each square represents a census site with 1 km square.

The field survey was conducted similar to previous studies in the Saga Plain in 1969 (Kubo and Matsuo 1972) and 2008 (Bussaka 2009). We established five census areas named Saga, Ogi (mentioned as Ushizu in the previous studies), Fukudomi, Chikugo‐riverside, and Kanzaki. A total of 70 square sections (1 km square) were established as shown in Figure 1, that is, 24 in Saga, 21 in Ogi, 7 in Fukudomi, 10 in Chikugo‐riverside, and 8 in Kanzaki. The plots were set up in similar locations to those in previous studies. In addition, 9 and 6 census areas with 1 km square were set up in Karatsu City and Kashima City, respectively, where nesting density is considered to have increased in recent decades (Saga Prefecture Board of Education 2014) (Figure 1).

### 2.3 Nesting Density and Nesting Locations

In Saga, magpies begin nesting earliest in late October of the previous year and reach their peak around February (Eguchi 2016). Then they lay eggs from March to April. Thus, it is expected that all nesting pairs will be active from late March onward. On the other hand, Eguchi (2016) pointed out that the detection rate of magpie nests formed in tree branches decreases from mid‐April when deciduous trees open their leaves. Therefore, the period from late March to early April is considered the best season to accurately identify the actual nests used by the magpie pairs. For this reason, our field investigations were conducted from March 30 to April 7, 2019.

We looked throughout the census areas and recorded nesting sites (trees or man‐made structures). When nests were found, we observed breeding pairs in the vicinity and classified the nest conditions as either in use (for nesting or brood‐rearing) or not in use (abandoned in the process of nesting or old nests).

The magpie is known to build a few nests within a territory but eventually completes and uses only one of them (Eguchi 2016). In our survey, only nests in use were included in the comparison of nesting densities. In field investigations, magpie nests are easily identified because they are spherical in structure, and no other birds forming similar nests are distributed in Japan (Kogaito and Wada 2011).

### 2.4 Relationship Between Land Use and Nesting Density

In this study, we compared the relationship between the percentage of farmland and buildings in each census area and the number of nests in the 2008 (Bussaka 2009) and 2019 surveys (current study). The percentages of farmland (including rice paddies, fields, orchards, etc.) and buildings (including road, railway, and building) within the observation areas were calculated using the R package ‘raster’ on the basis of land use data obtained from the digital national land information site of the Ministry of Land, Infrastructure, Transport and Tourism (https://nlftp.mlit.go.jp/ksj/gml/datalist/KsjTmplt‐L03‐b.html), with a spatial resolution of 100 m. For each survey plot, we calculated the percentage of a land‐use type within a 1 km radius from the center of the plot. The land use data from 2006 and 2014 were used to analyze the number of nests in the 2008 and 2019 surveys, respectively. Because of limitations in the availability of land‐use datasets, we used the closest available years to the survey periods—2006 for the 2008 survey and 2014 for the 2019 survey. Additionally, the land‐use data were only available at a spatial resolution of 100 m. Because these data were aggregated to proportions within a 1 km radius around each survey plot, the analysis is robust to cell‐level inaccuracies and better reflects the heterogeneous landscapes relevant to magpie habitat use. Since the survey locations varied slightly between the two surveys, we identified matching survey sites by determining whether the center of each site between the two survey periods was within 500 m. If an observation point was located within 500 m of the nearest point, we treated those two points as equivalent.

### 2.5 Apparent Nesting Success Rate and the Number of Fledglings

The apparent nesting success rate and the number of fledglings were surveyed from January or February to June of 2011–2013. In this survey, we regarded the areas where the nesting density was more than 15 breeding pairs/km^2^ as high density sites and less than 7 breeding pairs/km^2^ as low density sites. The survey was conducted in two high‐density sites: Ohdo (N33°14.2′, E130°21.5′) in Morodomi‐Cho and Inuido (N33°10.0′, E130°18.6′) in Kawasoe‐Machi, Saga City, and five low‐density sites: Kamikuro (N33°17.6′, E130°19.5′) and Kinryu (N33°20.0′, E130°18.0′) in Kinryu‐Machi, Saga City, Shurita (N33°15.2′, E130°20.4′) in Kose‐Machi, Saga City, and Shiwaya (N33°21.2′, E130°22.5′) and Tsuru (N33°19.5′, E130°22.6′) in Kanzaki‐Machi, Kanzaki City.

Because magpie nests are often found on utility poles and it is illegal to climb these poles, we inferred the progress of nesting to fledging through the observation from the vicinity of the nest. The survey was conducted at nearly 10‐day intervals throughout the breeding season. Referring to previous studies (Eguchi 2016), the stages from nesting to fledging were classified as follows.

1. Nesting period: the magpie pair carries twigs to form the nest.
2. Egg‐laying period: one of the mates is always inside the nest, and only the other one leaves the nest.
3. Nurturing period: a period when both mates may be out of the nest at the same time and are observed actively carrying food to the nest. The chicks can be heard chirping from the nest.
4. Successful departure from the nest: when the mates and the chick are confirmed to have left the nest and landed in a nearby field or other location.

The number of fledglings is rather easily confirmed because they stay in the vicinity of their natal nests within 10 days after fledging (Eguchi 1995). On the basis of our observations, fledglings typically stayed within a 25 m radius from their nest in this period, and the distance is much shorter than that from the nearest neighboring nest (at least 80 m). As mentioned in Eguchi (1995), nests can be discovered easily before egg‐laying, but since the mortality of chicks was not constant throughout the breeding season, the apparent nesting success rate, the proportion of nests that successfully produced at least one fledgling, was used in this study.

### 2.6 Statistics

The effects of magpie density (high or low) and census year on the success rates of egg‐laying, hatching, and fledging were analyzed using a Generalized Linear Model (GLM) with a binomial distribution and a logit link function. In the model, magpie density, census year, and their interaction were included as the fixed effects. The effects of density (high or low) and census year on the number of chicks fledged among successful nests were analyzed using a GLM with a Poisson distribution and a log link function, after assessing the absence of overdispersion for the number of chicks fledged. To evaluate differences in nesting pairs and land use (farmland, building, and forest areas) between 2008 and 2019, we used the non‐parametric Wilcoxon rank‐sum test because of non‐normal data distributions and potential outliers. For each variable, the Wilcoxon rank‐sum test was conducted separately to determine whether the observed differences were statistically significant.

In this study, we employed Spatial Durbin Models (SDMs) and a Linear Model (LM) to examine the effects of farmland, buildings, and forest land use changes on nest occupancy between the periods 2008 and 2019. The response variable in the models was the change in the number of nests over that period, whereas the explanatory variables were the changes in farmland, buildings, and forest areas. Because nesting numbers were expected to exhibit spatial autocorrelation, we assessed the presence of spatial autocorrelation in nest number changes using Moran's I statistic prior to conducting further statistical analysis. As spatially autocorrelated site data cannot be treated as independent, we needed to use a model that accounts for this non‐independence. Specifically, we created spatial weight matrices to define the neighbor relationships among sites using the nb2listw function from the ‘spdep’ package (Bivand 2022), on the basis of the geographical coordinates of the survey locations. Then, Moran's I test was performed under randomization to evaluate the spatial clustering of nest changes. We next fitted the SDMs using the lagsarlm function from the “spatialreg” package (Bivand and Piras 2021). In these SDMs, we included both spatially lagged and standard (non‐spatial) predictors. Spatially lagged predictors incorporate information from neighboring sites, capturing how land‐use changes in surrounding areas can influence a focal site (spillover effects). By incorporating these lagged terms directly into the model, we can better account for the possibility that land‐use changes in one site may influence nest occupancy in adjacent sites.

The models were then used to calculate spatial impacts, including direct, indirect, and total effects, using the impacts function. This approach provided a comprehensive assessment of spatial processes influencing nest occupancy, allowing us to differentiate between localized (direct) and spillover (indirect) effects of land‐use changes. Partial plots were generated for important predictors, such as agricultural and building changes. For each predictor, a sequence of 100 values was generated, and 100 bootstrapped repetitions were used to calculate predicted nest change numbers on the basis of the SDM coefficients. These plots illustrate the effects of each land‐use variable while controlling for other factors, providing robust measures of uncertainty around the predicted effects. All analyses were performed using R Statistical Software (v4.4.0; R Core Team 2024).

### 2.7 Ethics Statement

As mentioned above, the magpie habitat in this study area has been designated a national natural monument by the Japanese government. This study was primarily based on field observations and did not involve direct interference with the magpies. The study was conducted in accordance with national laws regarding the protection of national natural monuments and wildlife.

## 3 Results

### 3.1 Nesting Density and Nesting Locations

**FIGURE 2:**
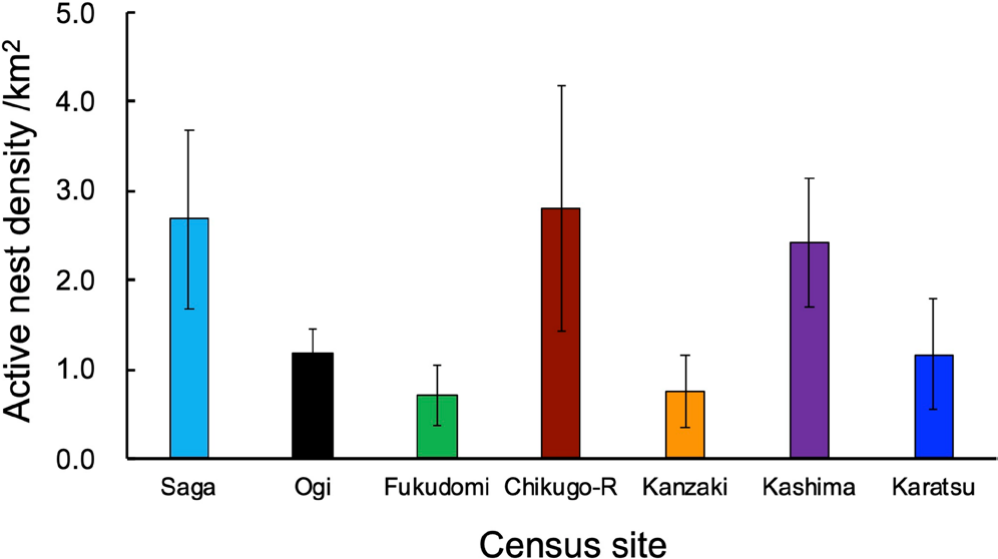
The mean number of active nests of magpies per 1 km square in Saga, Ogi, Fukudomi, Chikugo‐riverside (Chikugo‐R), and Kanzaki (see also Figure 1).

**FIGURE 3:**
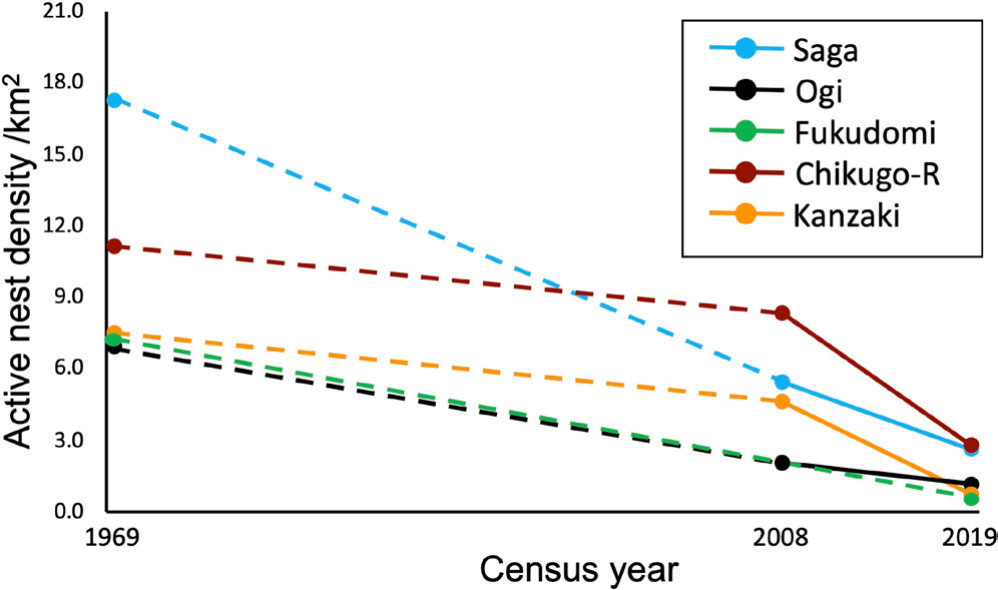
Trends of the mean number of active nests of magpies per 1 km square in Saga, Ogi, Fukudomi, and Chikugo‐riverside (Chikugo‐R), and Kanzaki (see also Figure 1). Since the magpie populations are known to have experienced a decrease in the early 1970s and an increase in the late 1970s (see text for details), the density trends between the 1969 and 2008 surveys are considered not linear. Thus, the trends are illustrated in dashed lines for this period.

The nesting density of magpies was relatively high in Saga, Chikugo‐riverside, and Kashima census sites and low in Fukudomi and Kanzaki census sites. The mean nesting density was less than 4.0 in all census areas (Figure 2). Comparisons with 1969 and 2008 survey data showed that the mean nesting densities were lower in 2019 than in the past at all census sites (Figure 3). The decline between 2008 and 2019 was particularly noticeable. Comparing the density of nests per survey plot in the Saga Plain, the number of plots with 11 or more nests decreased from 33.3% in 1969 to 6.6% in 2008 and 2.9% in 2019 (Table 1). On the other hand, the number of survey plots without nests increased from 6.3% in 1969 and 7.9% in 2008 to 34.8% in 2019 (the categories were followed Kubo and Matsuo 1972). Among 149 magpie nests found in the current survey, only one nest (0.7%) was formed on a tree, and the others were on man‐made structures; almost all of them were on utility poles, and a few were on other structures like steel towers. The ratio of magpie nests formed on trees was significantly lower (Fisher's exact test; *p* < 0.0001) in 2019 than in 2008, when 40 (13.0%) among 307 nests were on trees on the basis of Bussaka (2009).

**TABLE 1:** Frequency distribution of census areas with a different abundance of magpie nests in the Saga Plain.

Census year	The number of nests/km^2^		
	None	1–10	11 or more
1969	4 (6.3%)	38 (60.3%)	21 (33.3%)
2008	6 (7.9%)	65 (85.5%)	5 (6.6%)
2019	24 (34.8%)	43 (62.3%)	2 (2.9%)

### 3.2 Relationship Between Land Use and Nesting Density

**FIGURE 4:**
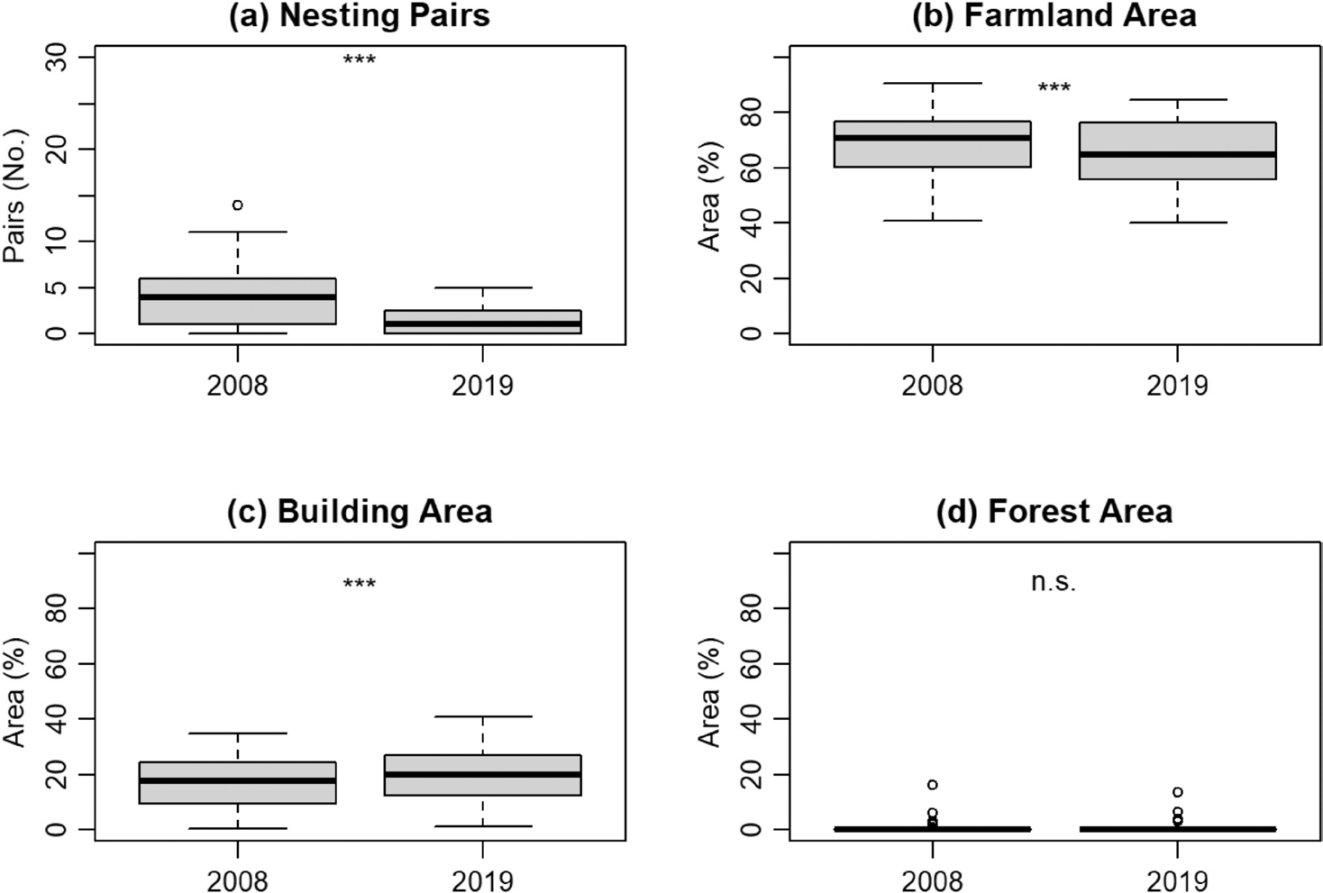
Box plots represent changes in (a) nesting pairs and the percentages of (b) farmland area, (c) building area, and (d) forest area between 2008 and 2019. The y‐axis shows the number of nesting pairs or the percentage of different land use types, whereas the *x*‐axis indicates the respective years. Statistical differences in each variable between years were assessed using the Wilcoxon rank‐sum test: ***p < 0.001 and n.s.: not significant.

In the areas where the surveys were conducted both in 2008 and 2019, the number of nesting pairs per survey site decreased from an average of 4.3 pairs in 2008 to 1.6 in 2019 (Figure 4a). In this period, the farmland area decreased from 69.1% to 64.9%, whereas the building area increased from 17% to 19.6% (Figure 4b,c). The forest area slightly decreased from 1.1% to 1.0%, but most survey sites did not contain any forest area (Figure 4d).

**FIGURE 5:**
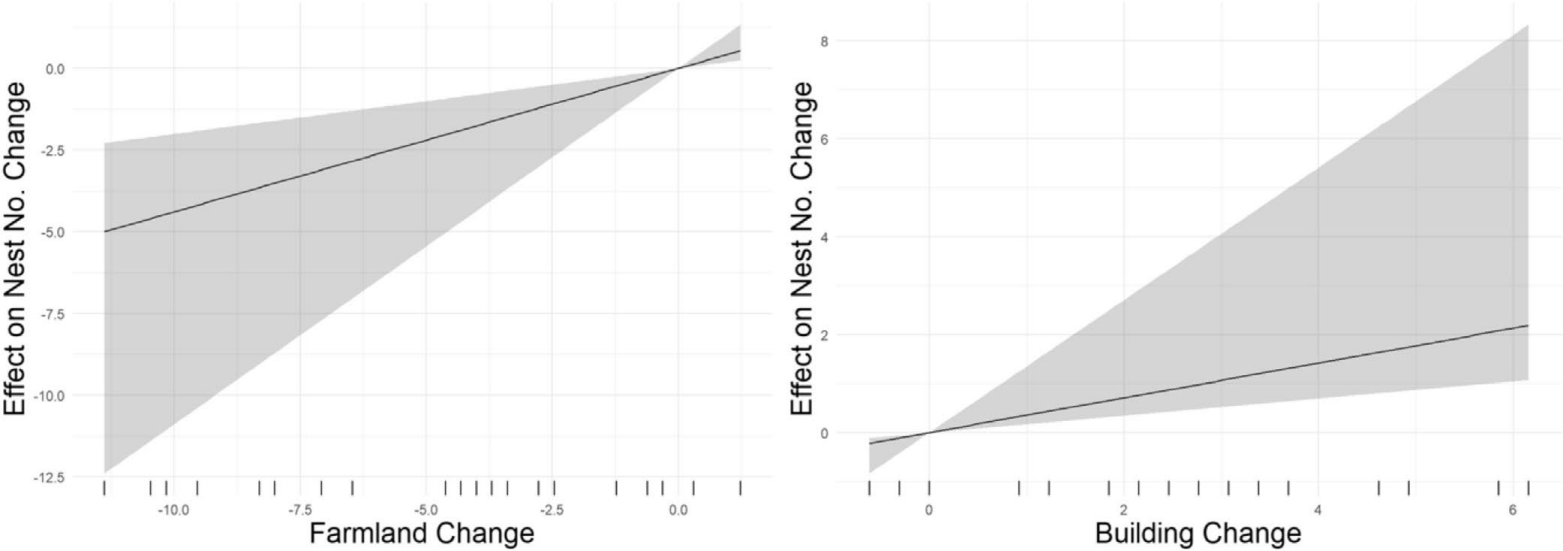
Partial Plots for Agricultural and Urban Changes (2008–2019 Period). The left panel shows the effect of agricultural changes on nest number changes, whereas the right panel shows the effect of urban changes. The shaded regions represent the 95% confidence intervals. The positive slopes indicate (1) a trend where decreases in agricultural changes are associated with decreases in nest number changes, and (2) urban expansion is associated with increases in nest number changes (both effects are borderline significant for the 2008–2019 period).

**FIGURE 6:**
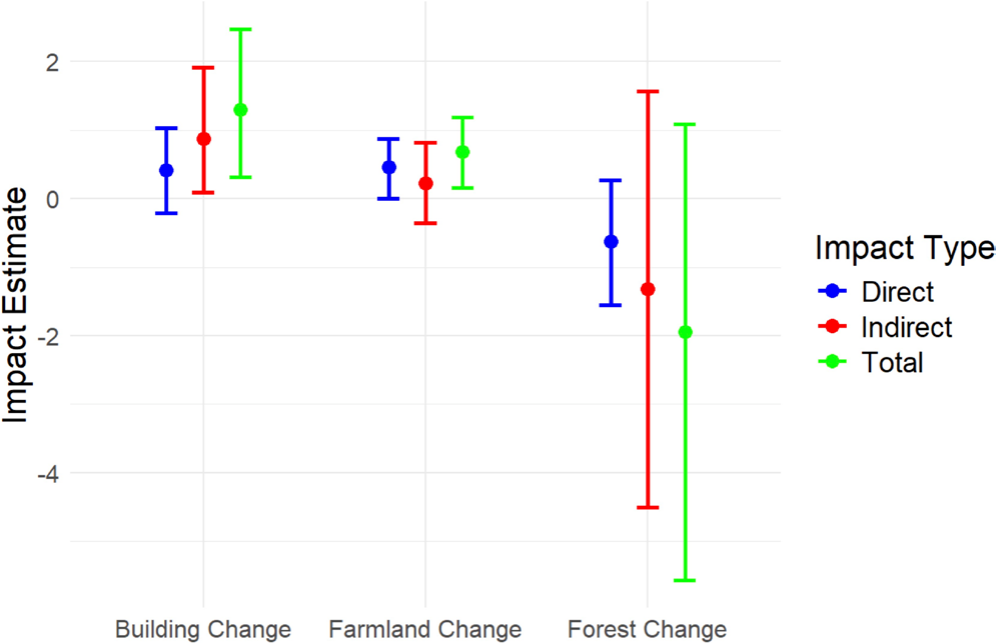
Impact measures with Confidence Intervals for the Durbin Model (2008–2019). The figure shows the direct (blue), indirect (red), and total (green) effects of changes in agricultural, urban, and forested areas on nest differences between the periods 2008–2019. Impact estimates are displayed with 95% confidence intervals.

The Moran's *I* test showed significant spatial autocorrelation in nest number changes (Moran's *I* = 0.546, *p* < 0.001). This means that locations with similar nest changes were geographically clustered. The SDM results (Table 2) showed that changes in farmland and building areas from 2008 to 2019 had marginally significant effects on nest number changes. The direct effect of farmland changes was 0.439 (*p* = 0.067), whereas building expansion had a marginally significant indirect (lagged) effect of 0.636 (*p* = 0.086). In contrast, changes in forested areas did not show significant effects. Partial plots for farmland (Figure 5, left) and building changes (Figure 5, right) confirmed these trends, with positive slopes indicating that building expansion was associated with increases in nest numbers, whereas decreases in farmland were associated with decreases in nest numbers (Figure 5). The overall model fit was evaluated using the LR test, which indicated that the spatial autocorrelation parameter (Rho = 0.236) was not significant (*p* = 0.224). The Wald test also suggested that the SDM does not perform substantially better than a non‐spatial model (*p* = 0.184). However, the SDM achieved a higher R‐squared value of 0.528, reflecting moderate model performance for the 2008–2019 period, compared to the LM model's R‐squared of 0.367, whereas both models showed close AIC values (164.474 and 165.186). Despite the lack of significance in the spatial parameters, these metrics suggested that the SDM better captured the variation in nest differences than the LM model, offering a more comprehensive understanding of the data. The impact analysis (Figure 6) further supported these findings by combining both direct and indirect effects as a total effect, leading to a stronger overall positive impact of farmland and building changes on nest numbers, even though individual effects in the SDM summary were only marginally significant. Direct, indirect, and total effects for farmland and building changes in the 2008–2019 period showed that building expansion had a higher impact on nest occupancy differences compared to farmland changes, with total effects being positive for both.

**TABLE 2:** Coefficients, standard errors, *z*‐values, and *p*‐values for direct and lagged effects of farmland, building, and forest land‐use changes from the Spatial Durbin Model and Linear Model (2008–2019). The response variable is the change in nest counts between two periods, whereas the explanatory variables are the corresponding changes in farmland, building, and forest areas, with “(Lag)” indicating spatially lagged values (indirect effects). The table also includes R‐squared, AIC, as well as Rho (spatial autocorrelation), and the Likelihood Ratio (LR) Test for model fit for the Spatial Durbin Model (for details, please refer to the results section).

Model	Spatial durbin model				Linear model			
Variable	Coefficient	SE	*z*	*p*	Coefficient	SE	*z*	*p*
(Intercept)	−2.572	0.945	−2.723	0.006	−2.194	0.937	−2.342	0.027
Farmland	0.135	0.074	1.831	0.067	0.189	0.056	3.343	0.002
Building	0.109	0.101	1.082	0.279	0.238	0.108	2.205	0.036
Forest	−0.163	0.142	−1.147	0.251	−0.262	0.16	−1.637	0.113
Farmland (Lag)	0.025	0.092	0.277	0.782				
Building (Lag)	0.196	0.114	1.718	0.086				
Forest (Lag)	−0.294	0.387	−0.759	0.448				
Rho (spatial autocorrelation)	0.236	0.177	1.328	0.184				
LR Test for Rho	1.478			0.224				
R‐squared	0.528				0.367			
AIC	164.474				165.186			

### 3.3 Nesting Success

**FIGURE 7:**
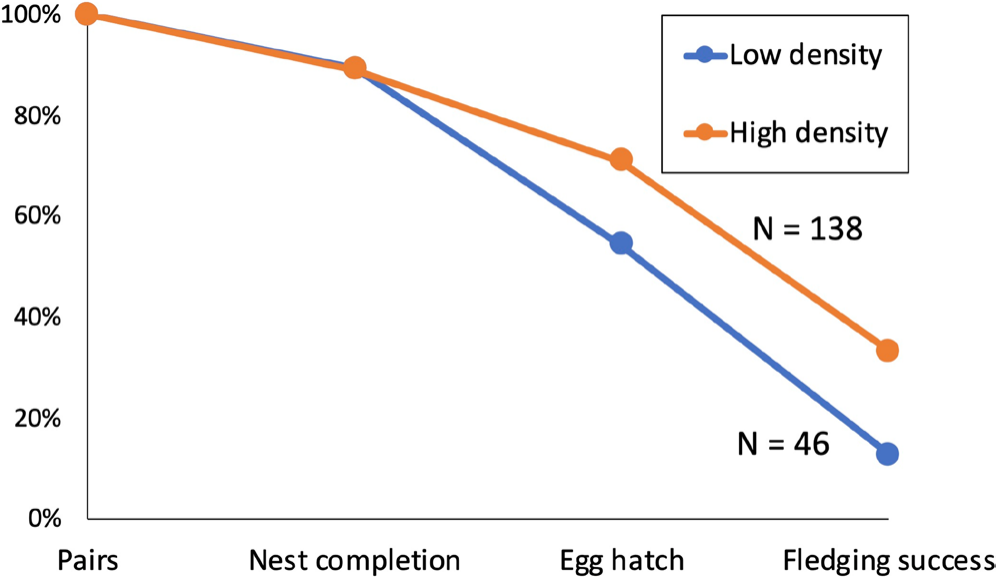
Breeding success of magpies in areas with high‐ and low‐density.

The number of breeding pairs was rather stable in all census sites between 2011 and 2013, 18–19 in Ohdo, 26–29 in Inuido, 5–6 in Kamikuro, 2–3 in Kinryu, 4 in Shurita, 0–1 in Shiwaya, and 2–3 in Tsuru. In total, we have observed 138 and 46 breeding pairs in high‐ and low‐density areas, respectively. There were no significant differences in the percentages of pairs that completed their nests between the high‐density and low‐density areas (Table 3) (Figure 7). The percentage of pairs that were considered to have hatched chicks was significantly lower in the low‐density areas (Table 3). The percentage of pairs whose chick(s) successfully left the nest was also significantly lower in the low‐density areas (Table 3). As a result, the percentage of pairs that fledged at least one chick was 33% and 13% among all pairs surveyed in the high‐density and low‐density areas, respectively. Among the successful pairs, the mean number of chicks fledged was not different, with 1.7 chicks in both areas (Table 3).

**TABLE 3:** Results of generalized linear models analyzing the effects of magpie density and census year on the reproductive success.

Event	Factor	χ^2^	Df	*p*
Nest completion	Density	0.000	1	1.000
	Census year	0.000	1	1.000
	Interaction	1.496	1	0.221
Chick hatch	Density	5.400	1	0.020*
	Census year	0.000	1	1.000
	Interaction	0.000	1	1.000
Successful leaving the nest	Density	4.500	1	0.034*
	Census year	0.491	1	0.484
	Interaction	0.113	1	0.737
Number of chicks fledged	Density	0.164	1	0.685
	Census year	0.069	1	0.792
	Interaction	0.080	1	0.743

* Asterisks indicate significant effects (*p* < 0.05).

## 4 Discussion

In this study, we clarified key aspects concerning magpie density, nesting sites, land use relationships, and reproductive success. First, magpie densities rapidly declined across most areas between 2008 and 2019, even as nesting on artificial structures significantly increased. Second, although both building and farmland areas positively influenced magpie density, the negative impact of farmland reduction outweighed the benefits of expanding building areas. Third, breeding success rates were notably higher in areas with high magpie density compared to those with low density.

### 4.1 Magpie Density and Nest Site

The nesting density of magpies in the Saga Plain was lower in 2019 than in 2008 in all areas surveyed. Although the magpie density was reported to have increased in recent decades in Kashima and Karatsu (Saga Prefectural Board of Education 2014), the density was still low in both areas in comparison with the density of the Saga Plain in 1969. Thus, at the whole population level of magpies in northern Kyushu, the increase in magpie density in these areas may not be sufficient to offset the rapid magpie decline in the Saga Plain.

As mentioned in the introduction, magpie populations recently prefer utility poles to trees for nesting sites, and nearly 90% of nests were formed on utility poles around 2010 (Bussaka 2009; Eguchi 2016). In our survey in 2019, the dependence of nest sites on utility poles was even greater, with more than 99% nesting on man‐made structures (almost all on utility poles). The shift in nest sites is consistent with broader patterns observed in urban‐adapted species, where several birds increasingly utilize artificial structures such as buildings, pipes, and bridges for nesting (Reynolds et al. 2019). In particular, the magpies have also been reported to nest on artificial structures such as chimneys and rooftops instead of trees in urban areas of Hangzhou, China (Wang et al. 2008; mentioned as Black‐billed Magpies *Pica pica* in the literature). On the basis of Eguchi (1996), the nesting success of magpies was approximately two times higher on utility poles than on trees. The nests on utility poles are often conspicuous, but magpies can repel the crows, a predator of their eggs and chicks, by direct attacks such as body blows. However, in tree nests, branches sometimes disturb the defensive behavior of magpies (Eguchi 1996). In addition, nests on utility poles are safe from ground predators such as cats (Eguchi 1996). Such differences may have contributed to the rapid spread of the habit of nesting on utility poles in magpie populations in the Saga Plain.

### 4.2 Relationship With Land Use

In terms of land use, the building area has increased, whereas the farmland area has decreased over the recent decade in the Saga Plain. Although both buildings and farmland areas had a positive effect on magpie density, the negative effect of the decrease in farmland areas was considered larger than the positive effect of the increase in building areas. Both the spatial Durbin model and linear regression model results demonstrate a strong correlation between changes in farmland and building areas and the decline in nesting pairs between 2008 and 2019. Areas that experienced greater reductions in farmland exhibited the largest declines in nesting pair numbers, suggesting that habitat loss due to agricultural reduction is a key driver of population decline. The positive correlation indicates that regions with more drastic reduction in farmland areas showed steeper declines, whereas areas with less reduction showed smaller population drops. Notably, changes in forest areas were not significant contributors to these patterns. This further highlights that urbanization and changes in farmland use were the dominant factors affecting nesting pair numbers during this period. However, the positive correlations with both farmland reduction and building expansion suggest more complex interactions at the landscape level, since most farmland should have been converted to buildings. In general, building expansion promotes the increase of utility poles with complex structures, where magpies prefer to make nests. In contrast, the conversion of farmland to buildings directly reduces foraging sites for magpies (Eguchi 2016). Thus, urban growth may act as a broader indicator of disturbance that could contribute to decline. These findings align with broader urban ecological patterns, where structural changes in the landscape may enhance nesting opportunities while simultaneously reducing foraging resources, leading to complex population responses (Shochat et al. 2006).

### 4.3 Spatial Spillover Effects

The spatial autocorrelation parameter (Rho) in the Spatial Durbin Model (SDM) was not statistically significant, indicating that the residual spatial structure may not have a significant influence on nesting pair decline. Nonetheless, the stronger explanatory power of SDM compared with the simple linear regression model suggests that SDM still explains a substantial proportion of the variance. This indicates that the land‐use variables with spatial structure remain important factors in explaining the observed patterns of decline. The impact analysis further emphasizes the importance of both direct and indirect effects of land‐use changes. The significant impacts of indirect and total building expansion, as well as direct and total farmland reductions, highlight the broader influence of urbanization and agricultural transitions on nesting patterns. Despite the non‐significance of spatial effects, these land‐use changes have both direct (localized) and indirect (spillover) effects, suggesting that broader landscape‐level disturbances are associated with local declines in nesting pairs, although redistribution beyond the study area cannot be ruled out.

### 4.4 Effects of Urbanization

As mentioned above, the decline in the number of magpies in the 1990s was more pronounced in areas with high building coverage (Saga Prefectural Board of Education 2014; Eguchi 2016). Urbanization since the 1990s has reduced the amount of agricultural land, which is available for foraging magpies, and this change is considered to result in a decrease in the density of magpies (Saga Prefectural Board of Education 2014; Eguchi 2016). In contrast, the decline in the 2010s was more conspicuous in areas with low building and high farmland coverages, and magpies are now found in high densities only in restricted areas with approximately a half‐building and half‐farmland ratio. The results of our current study suggest that the mechanism of magpie population decline is different between the 1990s and 2010s.

Kubo (1975) surveyed the life history of magpies in the Saga Plain and reported that the magpies establish communal roosts in bamboo groves and thickets near villages, with 50–300 individuals gathering from a distance of approximately a 2 km radius. In recent decades, large‐scale field development has progressed in rural areas, and the disappearance of groves and large trees, which results in the loss of roosting sites, has been noted there (Bussaka 2009). From the viewpoint of land use, the decrease of groves as roosting sites and of large trees as nesting sites is considered to have occurred in rural areas following the progress of field development. Because of such environmental changes, high densities of magpies may no longer be maintained in rural areas of the Saga Plain.

Recent large‐scale analyses of bird population trends in North America have revealed that the most severe declines often occur in areas where species are most abundant, including core habitats such as grasslands and aridlands (Johnston et al. 2025). These analyses suggest that even in rural landscapes, traditionally considered strongholds for many species, environmental changes such as land‐use conversion and climate stressors may be outpacing the adaptive capacity of bird populations. This pattern is consistent with our observation of magpie populations in the Saga Plain.

### 4.5 Reproductive Success

The proportion of magpie pairs that was presumed to have laid eggs was not significantly different between the high‐density and low‐density areas, suggesting that factors such as a lack of nesting materials were not relevant. In contrast, egg and chick survival was significantly lower in the low‐density areas than in the high‐density areas. The number of successfully fledged nestlings per pair did not differ between the high‐ and low‐density areas. The average number of young fledged in this study was 1.7, similar to or slightly lower than in previous surveys (average 2.2; Eguchi 1995; Eguchi and Takeishi 1997). These results suggest that success rates during the incubation and chick‐rearing periods are low in the low‐density areas.

Although mortality factors were not identified in our present study, predation, most notably by resident crows, is known to be the biggest cause of chick‐raising failure in magpie populations in the Saga Plain, accounting for more than 80% of failures (Eguchi 1995). In Saga City, the abundance of magpies was higher than that of resident crows in the 1990s (Saga Prefecture 1996), but recent investigations report an increase in resident crows (Bussaka 2009). In addition, several members of the Wild Bird Society of Japan, Saga, and local people have observed that resident crows predate magpie eggs and chicks (personal communications). In the past, the magpie has been known to display mobbing defense against crows. Such behavior becomes difficult in the low‐density areas with few neighboring nesting pairs, which may be related to the lower egg and chick survivals than in the high‐density areas.

## 5 Conclusion

In this study, we revealed that the magpie populations continue to decrease in the Saga Plain and their density rapidly declined in the 2010s. In terms of land use, both building areas and farmlands positively affect magpie densities, whereas the effects of farmland are greater than those of building areas, resulting in the magpie decline following the decrease in farmland areas. Egg and chick‐raising failures were significantly higher in areas with low magpie density than in those with high density. These results suggest that changes in land use patterns, as well as the increase in egg and chick mortality, affect the recent population decline of magpies in the Saga Plain. Further studies are needed to clarify the detailed cause of magpie population declines in the Saga Plain and future trends of magpie population dynamics there as well as in surrounding areas.

## Author Contributions

**Takuho Nagafuchi:** conceptualization (supporting), data curation (supporting), investigation (equal), writing – original draft (supporting). **Yasue Bussaka:** conceptualization (equal), data curation (equal), investigation (equal), methodology (equal), resources (equal). **Makihiko Ikegami:** conceptualization (supporting), data curation (supporting), formal analysis (equal), methodology (equal), software (equal), writing – original draft (supporting), writing – review and editing (supporting). **Kao Tsuchiya:** conceptualization (supporting), data curation (supporting), visualization (supporting), writing – original draft (supporting), writing – review and editing (supporting). **Makoto Tokuda:** conceptualization (lead), data curation (equal), formal analysis (equal), funding acquisition (lead), investigation (equal), methodology (equal), project administration (lead), resources (lead), software (lead), supervision (lead), validation (lead), visualization (lead), writing – original draft (lead), writing – review and editing (lead).

## Conflicts of Interest

The authors declare no conflicts of interest.

## Supporting information


**Data S1:** ece372193‐sup‐0001‐Data_S1.xlsx.

## Data Availability

All data generated or analyzed during this study is included in this published article or in Supporting Information files.
